# Myocardial late gadolinium enhancement cardiovascular magnetic resonance in patients with cirrhosis

**DOI:** 10.1186/1532-429X-12-47

**Published:** 2010-08-13

**Authors:** Dirk Lossnitzer, Henning Steen, Alexandra Zahn, Stephanie Lehrke, Celine Weiss, Karl Heinz Weiss, Evangelos Giannitsis, Wolfgang Stremmel, Peter Sauer, Hugo A Katus, Daniel N Gotthardt

**Affiliations:** 1 Department of Internal Medicine III, University Hospital of Heidelberg, Im Neuenheimer Feld 410, 69120 Heidelberg, Germany; 2 Department of Internal Medicine IV, University Hospital of Heidelberg, Im Neuenheimer Feld 410, 69120 Heidelberg, Germany

## Abstract

**Background:**

Portal hypertension and cardiac alterations previously described as "cirrhotic cardiomyopathy" are known complications of end stage liver disease (ELD). Cardiac failure contributes to morbidity and mortality, particularly after liver transplantation and transjugular intrahepatic portosystemic shunt (TIPS). We sought to identify myocardial tissue characterization and evaluate cardiovascular magnetic resonance (CMR) for diagnosis of cardiac impairment.

**Results:**

Twenty ELD patients underwent CMR for morphological, functional and tissue characterization by late gadolinium enhancement (LGE). Based on extent of LGE, patients were dichotomized into high and low LGE groups and analyzed regarding liver, cardiocirculatory and renal functions. CMR demonstrated hyperdynamic left ventricular function and a patchy pattern of LGE of the myocardium to a variable extent (range 2-62%) in all patients. There were no significant differences in Model for End-Stage Liver Disease (MELD), Child-Pugh score or the left ventricular ejection fraction between high and low LGE groups. QTc-interval was prolonged in 25% of the patients. E/A ratio was at the upper limit of norm; no difference between groups. Patients showing high LGE had a higher CI (p < 0.05). Biomarkers of myocardial stress were elevated. While NT-proBNP and c-Troponin-T showed no differences, PLGF and sFLT1 were lower in the high LGE group.

**Conclusion:**

CMR shows myocardial involvement in patients with ELD resembling appearance of myocarditis. The hyperdynamic circulation in portal hypertension may be an important factor. Larger prospective trials are warranted to confirm the association with severity and outcome of liver disease and to test the predictive power of CMR for patients listed for liver transplantation.

## Background

End stage liver disease (ELD) regularly leads to major alterations in the regulation of the cardiovascular system. Portal hypertension and/or hormonal changes in ELD induce a hyperdynamic circulatory state characterized by arterial hypotension and tachycardia and are often accompanied by ascites and electrolyte disturbances. Recent data also emphasizes the impact of liver function on renal (hepatorenal syndrome) and pulmonary (hepatopulmonary and portopulmonary syndrome) circulation [1], [2]. Driven by the high morbidity and mortality, significant research has been devoted to the causes and treatment of renal and pulmonary manifestations. Contrary to these efforts, little data is available on myocardial changes due to ELD. Although the hypothesis of ethanol-induced cardiomyopathy has been around for several decades, the role of the heart in the hypercirculatory state itself has only been addressed in a small number of studies [3], [4], [5]. Apparently, the hypercirculatory state was considered as proof of normal contractile function of the heart. A decreased ejection fraction is found only in a minority of cases with ELD. Some researchers propagate a specific disease entity called "cirrhotic cardiomyopathy" for the cases of advanced reduction of ejection fraction and for more subtle evidences of functional impairment such as prolonged QTc-interval, impaired response to physical activity/stress, reduced heart rate variability, or altered E/A in echocardiography eventually resulting in higher morbidity and mortality pre- and post-OLT[6], [3]. The improved survival of patients with ELD after orthotopic liver transplantation (OLT) and the hypercirculatory state after OLT and transjugular intrahepatic portosystemic shunt (TIPS) have directed research interests towards the myocardium [7], [8], [9].

Cardiovascular magnetic resonance (CMR) has become the gold standard method for the assessment of cardiac morphology and function in various cardiomyopathies (CMP) [10]. Areas of high signal intensity appearing 10-15 min after injection of the intercellular contrast agent gadolinium were first described as late enhancement (LGE) in regions of myocardial scarring after myocardial infarction [11]. This technique allows an additional, excellent imaging modality for the analysis of intercellular matrix. LGE can be detected in post-infarct scars, inflammation, as well as non-ischemic CMP, cardiac neoplasm and storage diseases such as amyloidosis. Typical LGE patterns have been defined for each of these diseases [12]. Moreover the extent of LGE is associated with the amount of affected myocardial tissue, e.g. in myocarditis or ischemic cardiomyopathy [13]. CMR is the preferred test for the repeated evaluation of disease course due to the lack of radiation and its non-invasive character [14].

Here we used CMR and serological markers for the evaluation of functional myocardial changes, non-invasive tissue characterization and the identification of specific cardiac lesions in cirrhotic patients listed for liver transplantation.

## Methods

### Patients

From March 2007 until July 2008, 20 consecutive patients with ELD had a CMR scan at the department of cardiology of the University of Heidelberg, Germany. All patients had been referred by the department of gastroenterology of the University of Heidelberg, Germany for evaluation of cardiac functional analysis. Exclusion criteria were decreased kidney function (glomerular filtration rate below 30 ml/min/1.73 m^2^) and general contraindications to CMR (e.g., metal implants, pacemaker, and claustrophobia). All procedures used in this study complied with the Declaration of Helsinki, were approved by our local ethics committee, and all patients gave written informed consent.

All patients were listed for liver transplantation. Two were waiting for re-transplantation due to chronic transplantation failure. Clinical characteristics are shown in Table 1. The mean age was 52.4 ± 9.4 years. The etiology of cirrhosis was alcohol in 12 patients, virus infection in three, autoimmune disease in three, and cryptogenic in two patients, no patient suffered from malignant disease. Gender distribution was 11 male and 9 female. The median Child-Pugh score was 9 (range 5-11) and the mean Model for End-Stage Liver Disease (MELD) score was 14.9 ± 6.3. No patient revealed clinical signs of impaired left ventricular dysfunction except one who had been diagnosed as "alcoholic cardiomyopathy" and showed moderately impaired left ventricular function. All patients were on therapy with non-selective beta-blockers (Propanolol).

**Table 1:** Patient characteristics

Parameter (n = 20)	Cirrhosis patients	Controls
Age (mean ± SD) [years]	52.4 ± 9.4 (24-68)	42.0 ± 12.9 (24-69)

Body mass index (mean ± SD) [kg/m^2^]	25.6 ± 4.9 (16.9-34.0)	24.2 ± 3.3 (18.9-33.0)

Sex (M/F)	11/9	60/60

Bilirubin (mean ± SD) [mg/dL]	4.68 ± 6.7 (0.33-28.8)	N/A

Albumin (mean ± SD) [g/L]	30.34 ± 7.2 (19-46)	N/A

MELD score (mean ± SD)	14.9 ± 6.3 (6.4-28.5)	N/A

Child-Pugh score (median)	9 (5-11)	N/A

Etiology		
Alcoholic	12	
Non-alcoholic	8	
Autoimmune	3	
Viral	3	
Cryptogenic	2	

MELD score Model for End-Stage Liver Disease; Range is in brackets, SD standard deviation, N/A not assessed

Routine echocardiography was performed on a Philips ie33 echo system (Philips Medical Systems, Netherlands). Doppler ratio of early to late transmitral flow velocity (E/A) was calculated.

### Healthy reference group

The hemodynamics of ELD patients were compared with a healthy reference group of 120 volunteers within our center. Subjects were considered as healthy (mean age was 42.0 ± 12.9 years) when no severe diseases were found in their medical history and a clinical examination, routine blood parameters, ECG, blood pressure measurements, spirometry as well as dobutamine stress and LGE CMR were within a normal range..

### CMR

All images were acquired with a 1.5 T magnetic resonance imaging system (ACHIEVA, Philips Medical Systems, Netherlands), a flexible cardiac five-element phased array coil and a vector electrocardiogram for R wave triggering using a standard MRI imaging protocol.

In brief, multiple short axis (SAX) cine images using a steady state free precession sequence with parallel imaging (balanced Fast Field Echo (FFE); Repetition time (TR)/Echo time (TE) = 2.9/1.45 ms; reconstructed voxel-size = 1.5 × 1.5 × 8 mm acquisition; sensitivity encoding (SENSE)-factor = 2) were acquired after the acquisition of true two- and four- chamber planes for the assessment of left ventricular ejection fraction (LVEF %).

A 2D FFE multi-slice SAX inversion recovery sequence (TR/TE = 2.9/1.0; reconstructed voxel-size = 2.0 × 2.0 × 5 mm acquisition; SENSE-factor = 2; TI = 200 - 280 ms) 10 minutes post-Gd (Gd = 0.2 mmol/kg of gadopentetate dimeglumine (Schering, Berlin, Germany)) was employed for the measurement of LGE.

**Figure 1:**
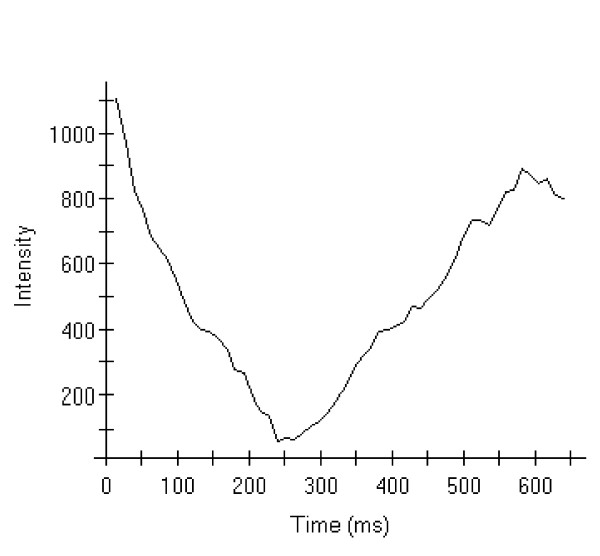
**T1 Mapping intensity graph based on a lock locker sequence to quantify the optimal inversion time for LGE imaging**.

The scan protocol was carried out in the following order: after a survey and reference scan, balanced cine FFE were acquired in 2-chamber, 4-chamber, short axis (multi-slice), and 3-chamber orientations. LGE scans were carried out in SAX, 4-chamber, and 2-chamber orientations 10 minutes after the injection of gadolinium. The inversion time was chosen very carefully by performing a T1 mapping using a lock locker sequence in each patient to estimate the correct inversion time (Fig. 1). The evidence of LGE was confirmed by imaging different views of the myocardium (i.e. 4 chamber view, 2 chamber view as well as short axis views).

### Image analysis

All CMR images were analyzed on a commercially available Cardiovascular Magnetic Resonance workstation with standard software (Philips Viewforum, Version 5.0, Best, Netherlands) by consensus reading of two blinded experienced observers.

### Definition of global LV function

For cine imaging, a modified 16-segment model according to the AHA definition [15] for the left ventricle was used to analyze left ventricular function and delayed enhancement per segment.

LV global function were calculated by the Philips View Forum software, version 5.0, after the manual tracing of the endocardial contours of the left ventricular wall at end diastole and end systole on the short axis cine data set.

### Analysis of late gadolinium enhancement

**Figure 2:**
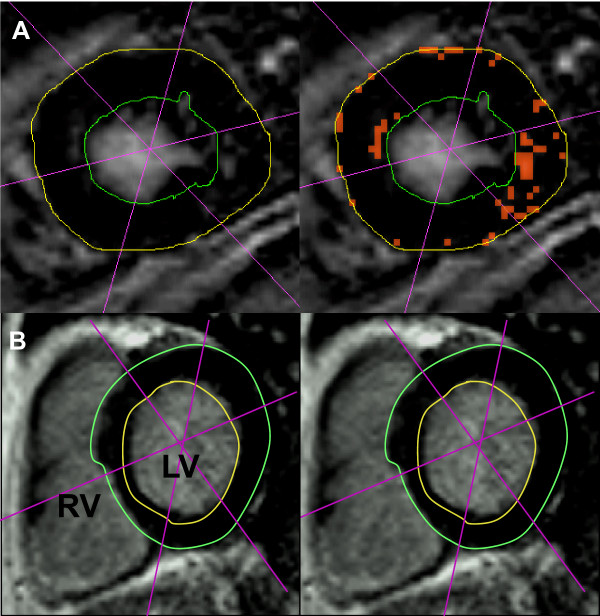
**LGE quantification in short-axis orientation in ELD patients with low LGE (detected by orange dots, 11% of myocardial mass (**A**) and a normal subject without delayed enhancement (**B**)**. LV = left ventricle, RV = right ventricle, S = interventricular septum, * p < 0.05

Segmental enhancement was quantified by the Philips View Forum Software 5.0 in all 16 segments and declared as a percentage of LGE of LV mass (Fig. 2). Signal intensity was measured as mean signal intensity plus standard deviations of mean (SD) for intra-individual analysis. Enhancement was defined as a signal intensity greater than five SD above the acquired mean signal intensity level [16].

To evaluate the association of late enhancement with the indices of cardiovascular stress or with indices of liver disorders, we dichotomized the patients in two equally large groups in respect to the percentage of myocardium with late enhancement.

### Clinical scores and blood tests

Blood sampling was carried out on the same day of the MRI scan. Blood samples were sent to the central laboratory of the University Hospital Heidelberg for routine parameters. Plasma aliquots were frozen at -80°C until biomarker assays were performed. High sensitivity Troponin T (hsTNT) was measured with a pre-commercial assay by electrochemo-luminescence methods (Roche Diagnostics, Mannheim, Germany).

Cardiac Troponin T (cTNT) was measured using the 4^th ^generation commercial one-step electrochemiluminescence assay based on electrochemoluminescence technology (Elecsys 2010, Roche Diagnostics, Mannheim, Germany).

N-terminal prohormone brain natriuretic peptide (NT-proBNP) was measured using a highly sensitive and specific electrochemluminescence immunoassay (Elecsys proBNP, Roche Diagnostics, Mannheim, Germany). The measurement range extends from 5 to 35,000 pg/mL. The minimal detectable concentration is 5 pg/mL and the coefficient of variation is 5.7% at 64 pg/mL.

PLGF and sFLT1 were measured using Elecsys^® ^PLGF (measurement range extends from 3 to 10,000 pg/mL) und Elecsys^® ^sFLT1 (measurement range extends from 10 to 85,000 pg/mL) routine assays by Roche Diagnostics, Mannheim, Germany.

### Statistical analysis

All statistical analyses were performed using SPSS 16 (SPSS Inc. Chicago, Illinois). Parametric (t tests) and non-parametric (Mann-Whitney U test) tests were used for continuous data. In addition, the Fisher exact test was used where applicable. All p < 0.05 were considered statistically significant.

## Results

### Evidence of hyperdynamic circulation in ELD patients

Hemodynamic parameters measured by CMR of the patients are shown in Table 2 and were then compared to data of the healthy reference group. Values are given as mean and +/- standard deviation: Heart rate was 74.9 ± 11.9/min (reference group 64.8 ± 9.4/min, p <0.01) and stroke volume 107 ± 20.8 mL (reference group 91.7 ± 30.3 mL, p = 0.08). Left ventricular ejection fraction (LVEF) was 72 ± 7.5% with a range from 49% to 83% (reference group 65.8 ± 6.96% with a range from 32.8% to 84%, p < 0.01). Cardiac output was 7.9 ± 1.63 L/min with a range from 4.4 to 11.1 L/min (reference group 6.7 ± 1.6 L/min with a range from 3.5 to 12.5, p < 0.01). The resulting CI yielded 4.36 ± 0.86 L/min/m^2^, ranging from 3.41 to 6.48 L/min/m^2 ^(reference group 3.6 ± 0.7 L/min/m^2^, ranging from 2.1 to 6.3 L/min/m^2^, p <0.001). Left-ventricular end-diastolic volume was 150.0 ± 35.4 mL (reference group 149.4 ± 33.8 mL, p = 0.91). These changes were in line with previous observations of hyperdynamic circulation as part of the altered cardiovascular regulation in patients with ELD.

**Table 2:** Hemodynamic characteristics

Parameter/mean ± SD	Cirrhosis patients	Controls	p
Late enhancement [%]	27 ± 16.5 (2-62)	N/A	N/A

Cardiac index [L/min/m^2^]	4.36 ± 0.86 (3.41-6.48)	3.6 ± 0.74 (2.10-6.31)	< 0.01

Ejection fraction [%]	72 ± 7.5 (49-83)	66 ± 7.0 (33-84)	< 0.01

Stroke volume [mL]	107 ± 20.8 (81-154)	97.4 ± 20.5 (53-155)	0.08

Cardiac output [L/min]	7.9 ± 1.63 (4.4-11.1)	6.7 ± 1.64 (3.5-12.5)	< 0.01

Heart rate at rest [bpm]	74.9 ± 11.9 (50-104)	64.8 ± 9.4 (45-91)	< 0.01

Syst. blood pressure rest [mmHg]	109.8 ± 14.4 (92-132)	126.4 ± 11.5 (105-160)	< 0.01

Diast. blood pressure rest [mmHg]	61.2 ± 7.8 (44-76)	76.4 ± 9.0 (46-104)	< 0.01

LVEDV [mL]	150.9 ± 36.4 (98-221)	149.4 ± 33.8 (87-252)	0.91

LVEDV left ventricular enddiastolic volume; Range is in brackets; Mann-Whitney-U test for differences was performed

### LGE CMR

**Figure 3:**
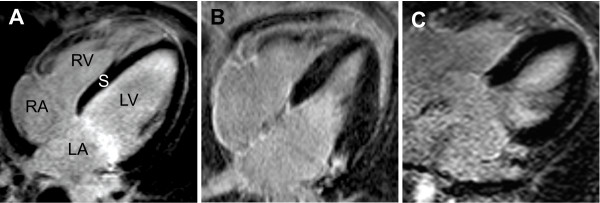
**LGE imaging in a normal subject who showed no signs of late enhancement in a four-chamber view (A)**. Patient suffering from ELD. Patchy pattern of delayed contrast enhancement (B and C). LA = left atrium, RA = right atrium, LV = left ventricle, RV = right ventricle, S = interventricular septum

We found variable degrees of LGE in all patients examined, indicative of myocardial injury despite hypernormal circulatory indices (Fig. 3). The amount of LGE measured in percentage of myocardium revealed a mean of 27% ± 16.5% and showed a patchy pattern similar to patients with myocarditis [17]. The minimum amount was 2%, the maximum as much as 62% (Table 2), the median was 25%. The detection of these changes was independent of the etiology of liver disease and was observed in patients with alcohol-induced, virus-related, autoimmune or cholestatic hepatic disease. No LGE was found in the reference group.

### Relationship of LGE with clinical indices of liver disease

We dichotomized the patients regarding the percentage of myocardium with late enhancement resulting in a high LGE group and a low LGE group (Fig. 2). We examined whether the parameters of liver disease were significantly different between the groups. However, there was no significant difference between the high and low LGE groups regarding MELD and Child-Pugh scores, nor single components of these composite scores (Table 3).

**Table 3:** Parameters of liver disease

Parameter	Low LGE	High LGE	p (Mann-Whitney U)
MELD (mean ± SD)	15.7 ± 5.2	12.3 ± 4.7	0.15

Child-Pugh Score (median; range)	10; (7-11)	8; (5-11)	0.12

Bilirubin (mean ± SD) [mg/dL]	4.4 ± 5.3	2.1 ± 1.4	0.40

Creatinine (mean ± SD) [mg/dL]	1.0 ± 0.5	1.1 ± 0.6	0.87

INR (mean ± SD)	1.46 ± 0.38	1.15 ± 0.16	0.06

Age (mean ± SD) [years]	50.9 ± 14.0	53.3 ± 6.0	1.00

MELD score Model for End-Stage Liver Disease, INR International normalized ratio for Quick value; Range is in brackets, SD standard deviation

Since alcohol is regularly considered to impair cardiac function and alcoholic cardiomyopathy is a known complication of chronic alcohol consumption, we analyzed the association of the etiology of cirrhosis and the extent of LGE. In our cohort, a high LGE could be found in a significantly higher extent in the alcoholic cirrhosis group than in the non-alcoholic cirrhosis group (Table 4).

**Table 4:** LGE vs alcoholic etiology

N	Non-alcoholic cirrhosis	Alcoholic cirrhosis
Low LGE	5	2

High LGE	1	7

P = 0.041 (Fisher Exact Test), LGE late gadolinium enhancement

### Extent of LGE

**Figure 4:**
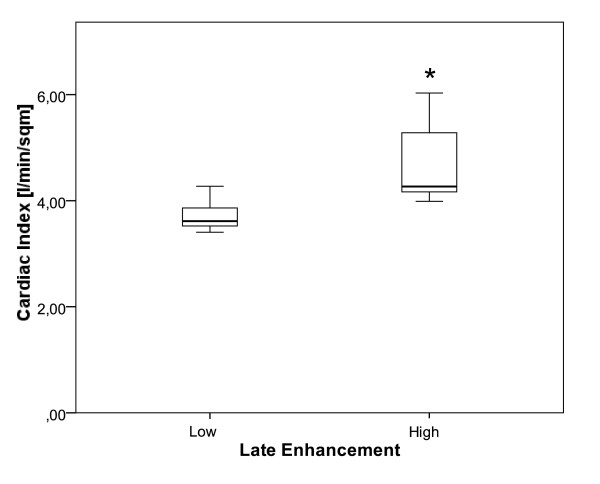
**Box plots of CI data are shown**. The more pronounced hyperdynamic circulation in the high LGE group (right) compared to the low LGE group is depicted (p = 0.029).

We analyzed whether the amount of LGE was associated with functional parameters of the cardiovascular system in the low and high LGE groups. There were no significant differences in stroke volume, ejection fraction, heart rate, and systolic and diastolic arterial blood pressure between these two groups (data not shown). Since hyperdynamic circulation to a varying degree was found in all patients in out study group, we examined the cardiac output, CI and presence or absence of hyperdynamic status defined as a Cardiac Index (CI) of greater than or equal to 4 L/min/m^2^, or less than 4 L/min/m^2^, respectively. Cardiac output showed a trend (p = 0.073) and CI (p = 0.014, Fig. 4) and hyperdynamic status (p = 0.029, Table 5) were significantly different between the low and high LGE groups. To evaluate established indicators for cirrhotic cardiomyopathy in our cohort we analyzed QTc-interval and signs of impaired diastolic function on echocardiography. Though QTc-interval was prolonged in 25% of the patients, E/A ratio by echocardiography was normal in all patients.

**Table 5:** LGE vs CI

N	CI < 4 L/min/m^2^	CI ≥ 4 L/min/m^2^
Low LGE	6	1

High LGE	1	6

P = 0.029 (Fisher Exact Test), LGE late gadolinium enhancement

### Serological markers of cardiomyopathy

NT-proBNP and hsTroponin T are established serological markers of ischemic and non-ischemic CMP and serum levels of these proteins were measured and these values were markedly increased in these patients (Table 6), while cTNT was within normal ranges.

**Table 6:** Established and potential serological markers of cardiomyopathy

Marker (mean ± SD)	Low LGE	High LGE	P (Mann-Whitney U)
NT-proBNP [ng/L]	125.3 ± 82.2	101.8 ± 64.1	0.18

hs-TNT [ng/L]	5.6 ± 4.2	7.4 ± 3.8	0.56

sFLT1 [ng/L]	143.7 ± 22.6	77.1 ± 21.2	0.06

PLGF [ng/mL]	36.8 ± 10.3	20.6 ± 2.2	0.02

hsTNT high sensitivity cTNT, LGE late gadolinium enhancement, NT-proBNP N-terminal prohormone brain natiuretic peptide, hsTNT high sensitivity cTNT, sFLT1 soluble short form of vascular endothelial growth factor receptor-1, PLGF placental growth factor

**Figure 5:**
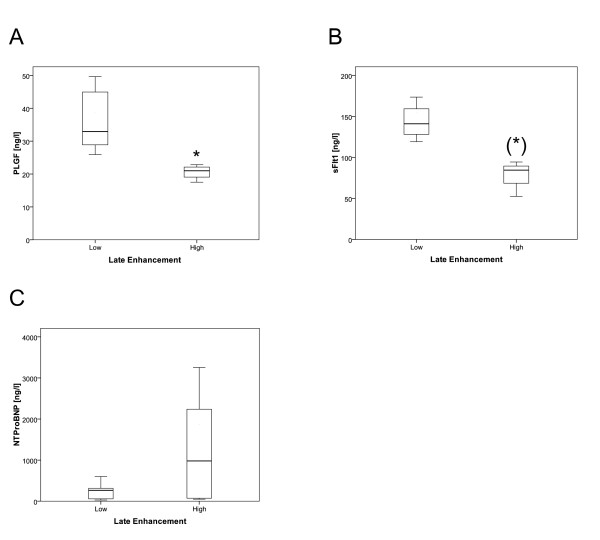
**The differences in serological markers of CMP are shown as box plots**. (**A**) PLGF was significantly decreased in the high LGE group (p = 0.016). (**B**) sFLT1 showed a strong trend to be decreased in the high LGE group (p = 0.057) (**C**) NT-proBNP was not significantly different between the two groups, but very high values ( > 1000 ng/L) could only be found in the high LGE group, * p < 0.05

We measured other biomarkers which have been associated with cardiomyopathy; of these, PLGF showed a significant correlation and sFLT1 showed a strong trend. Both were decreased in the high LGE group (Fig. 5).

## Discussion

Liver failure in patients with ELD was the major life limiting factor until OLT has become the therapeutic option of choice resulting in a major improvement of survival. Subsequently, other comorbidities have become more apparent and clinically relevant. The increasing rates of cardiovascular complications after liver transplantation have raised the question of the underlying reasons for heart failure and electrical abnormalities. The increasing number of patients who receive liver transplantation has conferred a substantial body of evidence for hemodynamic deterioration following surgery. Accordingly, rates of pulmonary oedema have been reported to occur in up to 56% of liver transplant recipients following surgery, and hemodynamically significant arrhythmias in 27%, and congestive heart failure in as many as 5.6% [18]. In a more recent study, 82/179 patients after OLT suffered from cardiac decompensation and cardiac causes were the leading cause of death in these patients [9]. Although adverse intraoperative cardiac events and history of cardiac disease are independent predictors of death due to cardiac cause, established risk assessment--including ECG, echocardiography, coronary angiography, and myocardial perfusion scintigraphy--fails to predict a complicated perioperative course or death attributed to cardiovascular events. A good diagnostic means for investigating cardiocirculatory changes before OLT is still lacking. For this reason, better detection of patients at risk for cirrhotic cardiomyopathy is required.

We therefore used CMR as the gold standard for the imaging of cardiomyopathies and biomarkers as well-established tools for the detection of heart failure. In line with previous findings, all patients in our study group showed a hyperdynamic cardiac function [4] (i.e. increased heart rate and cardiac output at rest suggesting healthy hearts). In contrast, NT-proBNP and hsTNT were elevated in those patients, indicating a concealed form of heart failure. One could hypothesize that these markers are evidence of a barely compensated cardiocirculatory system close to the edge of decompensation.

Since hsTNT is supposed to uncover minimal damages of myocardial tissue [19], we performed LGE CMR to detect potential myocardial injury. One major finding in this study is that CMR allows detection of LGE in patients with end-stage liver disease.

LGE was traditionally only considered to be associated with fibrosis due to ischemic heart disease [11] but was then also described in non-ischemic cardiomyopathies, in infiltrative disorders such as amyloidosis and Fabry's disease [12], More recent reports have suggested there is enhancement in regions of myocarditis [20]. LGE can therefore be caused by fibrosis or pathological deposits in the myocardium as well as an inflammatory process. Recent work could show that the pattern of LGE is indeed characteristic of different cardiac diseases, allowing for an even better diagnosis and etiological allocation [21,22].

Though LGE could be detected in patients regardless of etiology of liver disease, indicating a common mechanism originating from cirrhosis, the extent of LGE was more pronounced in patients with alcoholic liver cirrhosis. This supports the hypothesis of an additional modifier originating from alcohol abuse. The patchy pattern of LGE in our study group is comparable to the pattern found in acute myocarditis, where a partial reversibility of LGE in the chronic phase was described before [23]. However, patients with acute myocarditis show markedly elevated levels of conventional cTNT, which could not be detected in our cohort. The pattern differs from other non-ischemic CMP or after myocardial infarction, as mentioned before. Interestingly, there is a striking mismatch between the degree of LGE and the degree of LV function. Although a mean of 27% of the myocardial mass was affected by LGE, only one patient disclosed depressed systolic LV function. This indicates that the detected LGE in this study may not be due to fibrosis, but rather caused by pathological deposits (e.g., accumulated cardiotoxic metabolites, oxygen supply/demand mismatch due to arteriovenous pulmonary shunts, inflammation, a yet unknown mechanism). This might be explained by the findings of previous studies, which showed that cardiomyocytes and the trabecular network of the myocardium are changed in cirrhotic patients [3]. Histological specimens to examine possible alterations were not available in our patients.

In order to analyze correlations of established clinical parameters with LGE, we dichotomized our patient group, one with low amounts of LGE and one with high amounts of LGE. This resulted in a cut-off value for high LGE of approx. 25% affected myocardium. These two groups were then compared in regard to liver and cardiocirculatory functions. Interestingly, we could not find a significant difference in markers of liver function including MELD and Child scores between these two groups. An obvious link between liver function and LGE could therefore not be recognized. Nevertheless, we could demonstrate a significant difference regarding the CI between the low and high LGE groups. Cirrhosis leads to portal hypertension of varying extents. Complications of portal hypertension include the development of portosystemic collaterals ending among others in varices bearing the risk for variceal bleeding. Furthermore, portal hypertension results in peripheral and splanchnic vasodilatation and compensatory hyperdynamic cardiac function, which can be measured by an increased CI. As noted above there is a striking mismatch between LGE and LV function. In this context it might appear even more surprising that an even higher cardiac function as measured by cardiac index is associated with higher degrees of LE. This is vice versa to published data on LGE in other patient cohorts [24]. One could speculate that these circumstances result in a high cardiac workload burden and an incipient cardiac decompensation. Another explanation might be that detection of LGE in cirrhosis patients is caused by different mechanisms. And finally one can not exclude that the inclusion and exclusion criteria into this study might have influenced these outcomes. This would be in line with previous studies regarding cardiocirculatory regulation and hypercirculatory status in cirrhotic patients showing findings that have not yet been well explained [3]. In our study we could not find an association between LGE and other cardiac tests to be related to cirrhotic cardiomyopathy. Prolonged QTc-interval and reduced E/A ratio did not correlate with LGE. In fact the former could only be found in 25% of patients and the latter was normal in all patients. This might be attributed to the fact, that the aim of this study was not to examine patients with overt cardiac alterations, but detect common changes found in not preselected patients.

Since performing CMR might not be feasible in all ELD patients or contraindicated, we investigated several potential markers for cirrhotic cardiomyopathy. SFLT1 and PLGF were decreased in patients in the high LGE group. sFLT1 is the soluble short form of vascular endothelial growth factor receptor-1 (VEGFR-1), a member of the VEGFR family. It has been shown to interact with placental growth factor (PLGF). The involvement of both proteins in angiogenesis, the circulatory and renal dysregulation in preeclamptic patients, and more recently as part of the development of atherosclerosis have all been discussed [25], [26]. Most interestingly, VEGF, its receptors and PLGF have been implied in the regulation of neovascularization in portal hypertension. This implication not only points to new diagnostic but also to new therapeutic approaches [27], [28]. Based on these data, one might speculate that these factors also play a role in circulatory dysregulation in ELD patients. Additionally, these biomarkers might be instrumental in detecting patients at risk and in monitoring them over time. No differences in other potential biomarkers were observed between the two groups.

How might these data affect clinical practice? Cardiac complications are a frequent cause of morbidity and mortality in patients undergoing liver transplantation and TIPS. These two interventions pose a stress on the cardiocirculatory system and can lead to a decompensation of cardiac function. This occurs despite regular and careful previous cardiological evaluation [9]. To our knowledge, this is the first description of LGE and hsTNT elevation in patients with ELD. CMR and hsTNT as well as NT-proBNP might enable clinicians to detect myocardial alterations, which is usually hampered by the hyperdynamic LV function in these patients. Further and larger studies are needed to confirm our results and in addition allow for more subtle subgroup analysis and focus on outcome data. These could then lead to a new risk stratification for cardiac complications based on these new diagnostic assessments and subsequently to an adapted and optimized perioperative management or to a treatment of the myocardial alterations itself. Furthermore it could lead to the exclusion of patients from OLT or TIPS due to high risk of development of heart failure after these interventions.

The association of the degree of LGE and hyperdynamic status in portal hypertension that could be demonstrated warrants further investigation and might be the basis for future studies examining its pathophysiological backgrounds.

Since previous studies could demonstrate a normalization of the hemodynamic parameters after liver transplantation in ELD patients [29], further studies have to be done, including follow-up measurements investigating the extent of a possible reversibility of these myocardial alterations.

## Conclusions

Based on our findings, we conclude that myocardial alterations have a high prevalence among ELD patients, can be superbly detected and quantified by CMR and are linked to portal hypertension. Their exact role related to "cirrhotic cardiomyopathy" has to be determined. Due to its unique ability to provide non-invasive, highly sensitive tissue characterization combined with functional analysis, CMR may become the gold standard for the diagnosis of myocardial alterations and its severity as it is in other cardiac diseases. Future experimental and clinical work including outcome analysis is needed to clarify the mechanism and possible consequences for risk stratification and treatment, especially regarding patients undergoing TIPS or liver transplantation.

## Abbreviations

CI: Cardiac Index; CMP: Cardiomyopathy; CMR: cardiovascular magnetic resonance; CTNT: cardiac Troponin T; ELD: end-stage liver disease; FFE: Fast Field Echo; HSTNT: high sensitivity cTNT; INR: International normalized ratio for Quick value; LGE: late gadolinium enhancement; MELD: Model for End-Stage Liver Disease; NT-PROBNP: N-terminal prohormone brain natiuretic peptide; OLT: orthotopic liver transplantation; PLGF: placental growth factor; SAX: short axis; SENSE: sensitivity encoding; sFLT1: soluble short form of vascular endothelial growth factor receptor-1; TE: Echo time; TIPS: transjugular intrahepatic portosystemic shunt; TR: Repetition time

## Competing interests

E. Giannitsis and H. Katus have served as speakers for and received grants from Roche Diagnostics. H. Katus holds patents jointly for cTNT assay and hsTNT assay.

## Authors' contributions

DL study design, data acquisition and analysis, writing the manuscript. HS data analysis and writing the manuscript. AZ data acquisition and analysis, SL data acquisition, CW data acquisition and analysis, KHW data analysis, EG study design and writing the manuscript, WS study design and data analysis, PS data acquisition and analysis, HAK study design and writing the manuscript, DNG study design, data acquisition and analysis and writing the manuscript. All authors approved the final version of the manuscript.
